# Limb lengthening over a nail can safely reduce the duration of external fixation

**DOI:** 10.4103/0019-5413.41857

**Published:** 2008

**Authors:** Milind Chaudhary

**Affiliations:** Centre for Ilizarov Techniques; Govt Medical College, Akola - 444 001 and Jaslok Hospital, Mumbai, India

**Keywords:** Consolidation time, intramedullary nails, lengthening over nails, limb lengthening

## Abstract

**Background:**

Limb lengthening using Ilizarov external fixation is safe, but the consolidation phase tends to take too long. A method that can safely reduce the time spent in external fixation would help increase patient tolerance and comfort. We report our results of lengthening over nails (LON) method in which an interlocking nail was used along with an Ilizarov external fixator to reduce external fixation duration in limb lengthening. This is a retrospective study.

**Materials and Methods:**

Twenty-seven lengthening surgeries were done with the LON method in 23 patients with 22 tibiae and five femora during the last 12 years. Length gain ranged from 1.5 cm to a maximum of 9.8 cm with a mean of 4.6 cm. The mean modified Paley difficulty score was 7.6 points. Fourteen associated procedures were performed in these patients, including equinus contracture releases, supracondylar osteotomies, ilizarov hip reonstruction and ankle fusion. We had a 29% rate of complications which included one problem, three obstacles and four complications with no serious deep intramedullary infections. Our rate of complications compares favorably with series reported in the literature. External fixation duration was reduced significantly to a mean of 17.8 days per cm.

**Conclusions:**

A combination of intramedullary nailing along with external fixation significantly reduces external fixation time while maintaining low rate of complications. Great care needs to be taken to prevent pin track infection and deep intramedullary sepsis.

## INTRODUCTION

Limb lengthening with the Ilizarov technique is a safe and successful surgical procedure with well understood biological principles. The consolidation phase of lengthening can take twice as long as the distraction phase in children and as long as thrice or four times in adults.^1^ Patients constantly demand removal of the fixator after the distraction phase is over. The fixator cannot be removed till the cortices are well formed.^1^ Early removal is fraught with risks of loss of length as well as bending and axial deviation.^1^

Lengthening with a combination of an intramedullary nail entails locking the nail at the increased length at the end of the distraction phase and removal of the external fixator. The regenerate is protected by the nail in the consolidation phase and external fixation duration can be reduced by 40-60%. It was first performed by Bost and Larsen in 1956^2^ and has been popularized by Paley *et al*.^1^

We report our experience of a small series of limb lengthening performed over an intramedullary nail over the last 12 years.

## MATERIALS AND METHODS

Twenty-seven lengthenings over a locking intramedullary nail were performed in 23 patients over the last 12 years at our institute. Twenty-two tibial lengthenings and five femoral lengthenings were performed [Table 1]. These segments were chosen for lengthening over nails (LON) as they had no significant deformity that needed to be corrected at the same level simultaneously. The amount of lengthening achieved ranged from 1.5 cm to 9.8 cm with a mean of 4.6 cm. Percentage lengthening [Table 2] ranged from 4-31% with a mean of 14.8% of the initial length of the segment lengthened. Two of our patients had a very small amount of length gain of 1.5 cm and 2 cm which was only 4% of initial length. Both these patients suffered from polio and were tested before surgery to definitely improve their gait with a raise of this amount. They chose to have the LON procedure as it would take much less time in external fixation and necessitate fewer follow-up visits from far away.

**Table 1:** Details of patients and lengthening over nail (LON) procedure

Name	Age	Sex	T/F	Length	Level of corticotomy	Duration of exfix in weeks/days	Diagnosis	Complications	Associated procedures
YA	9	F	T	3.8	u3	10.2/72	Cerebral palsy		
AK	20	M	T	5	u3	8/56	Polio		Equinus correction
SC	25	F	T	4	u3	9.4/67	PM bowing	10° Procurvatum	
GT	30	M	F	6.8	u3	21/147	Polio		FFD correction
JC	22	M	F	7.5	u3	13/91	Growth arrest		
KJ	23	M	T	4.5	u3	10/70	PM bowing		
KT	19	M	T	9.8	u3	32/224	Polio	Equinus contr.	Ilizarov SCO
GL	20	M	T	1.5	m3	4.3/31	Polio		
ND	22	M	T	4.9	u3	8.1/57	Polio		
RD	28	M	T	3.5	m3	6/42	Polio		
VR	26	F	T	4	m3	17/119	Polio		Ilizarov SCO
GS	22	M	T	6.2	m3	12/84	Polio		Equinus
SK	23	M	T	6	L3	10/70	Polio		Ankle fusion
SK	23	M	F	2	m3	8/56	Polio		SCO with IM nail
BT	25	F	F	6	u3	14.3/101	Growth arrest	Premature consolidation	Repeat corticotomy persistent shortening 7 cm
PW	22	F	T	2.4	m3	10/70	Polio		Equinus correction
BS	19	M	T	3	u3	18/126	Polio		Equinus correction
MD	18	M	T	3.2	u3	12/84	Polio		Ilizarov SCO
HP	23	M	T	5.6	u3	14/98	Constitutional short stature	Int rotation	Bowing
HP	23	M	T	6	u3	14/98	Constitutional short stature		Bowing
SC	19	M	T	8.5	u3	20/140	Chondro metaphyseal dysplasia		
SC	19	M	T	8.5	u3	20/140	Chondro metaphyseal dysplasia	Varus def	TSF fixator for varus correction
HP	20	F	T	3.5	u3	10/70	Congenital		
NB	49	M	T	2	u3	4/28	Polio		
AY	22	M	F	2.5	m3	7/49	Growth arrest	Premature consolidation	Persistent shortening 8 cm
AY	22	M	T	7.3	u3	14/98	Growth arrest		
GW	24	M	T	6.4	u3	20/140	Hip dysplasia		Ilizarov hip reconstruction

SCO - supracondylar osteotomies, FFD - fixed flexion deformity, TSF - taylor spatial frame

**Table 2:** Table of patients with Paley's score of difficulty and percentage lengthening

Name	Paley score	Init length	% lenghtening
YA	4	22	17.20
AK	8	30	16.6
SC	5	35.5	11.2
GT	13	42	16.1
JC	9	32	23.4
KJ	6	38.5	11.6
KT	15	31.5	31.1
GL	4	32.5	4
ND	7	34.5	14.2
RD	6	38	9
VR	8	34	11.7
GS	9	35	17.7
SK	10	37.5	16
SK	6	42	4
BT	12	27.5	22
PW	6	33	7
BS	6	39	7
MD	6	34.5	9.7
HP	8	38	14.7
HP	8	38	15.7
SC	9	29.5	28.8
SC	9	29.5	28.8
HP	5	31.5	11
NB	6	39	5
AY	7	33	7.5
AY	11	34	21.4
GW	9	35	18.2

Fourteen lengthenings were performed for poliomyelitis, four for growth arrest caused by osteomyelitis in childhood, two each for chondro metaphyseal dysplasia, congenital posteromedial bowing of tibia and constitutional short stature and one each for shortening associated with congenital scoliosis (case 23), cerebral palsy (tibial shortening of 3.5 cm) and hip dysplasia caused by septic arthritis in childhood (case 27). Case 23 with congenital scoliosis had a true shortening of 2 cm and apparent shortening of another 1.5 cm which could not be compensated due to spinal fusion performed earlier. Hence she needed a 3.5 cm lengthening. Case 27 with hip dysplasia secondary to septic arthritis of childhood underwent Ilizarov hip reconstruction and the tibia was lengthened by 6.4 cm over a nail. Since lengthening takes a longer time to consolidate rather than the femoral osteotomies, we wanted to remove the femoral frame earlier to maintain full range of motion (ROM)in the knee. The shortening was compensated by lengthening the tibia. The mechanical axis was already corrected in the femur with the Ilizarov hip reconstruction procedure. Ages ranged from nine years to 49 years (mean 23 years). Most of the patients were between 18 and 30 years of age. Only two were outside this range; one was a nine-year-old child suffering from cerebral palsy (CP) and the oldest was 49 years of age [Table 1].

### Tibial Operative Procedure

The tibial lengthening surgery consisted of inserting an intramedullary (IM) nail after reaming to a diameter at least 1½ mm larger than the diameter of the chosen nail. The proposed corticotomy site was predrilled to allow the reamings to escape and not increase intramedullary pressure. The corticotomy was made a few cm below the location of the Herzog curve of the tibial nail. The length of the IM nail was chosen to fall short of the ankle joint by about 3 to 5 cm. The nail was locked at the proximal end and left unlocked distally. A fibular osteotomy was performed in the middle-lower third junction and a syndesmotic screw was inserted distally to ensure integrity of the distal tibio-fibular joint.

A standard locking IM nail was used in most patients. Patients with poliomyelitis with poor quadriceps strength have a lesser Herzog curve. A modified humerus nail with an angulation of 5 to 6° was used in all of these. Two interlocking screws were used proximally and distally in polio patients. For other indications, a design of nail with more number of interlocking screws was used proximally to prevent instability and deformation of the proximal fragment.

The nine-year-old child suffering from CP was the only skeletally immature patient and a specially designed locking Ender nail^3^ was used to gain entry below the physis and permit early removal of the fixator.

External fixation pins were passed with a clear margin of 3 to 5 mm from the nail. Proximally, one half pin was passed in the antero-posterior direction around the Gerdy's tubercle at a distance of about 1 cm lateral to the nail. One plain Ilizarov wire was passed 1 cm posterior to the nail. These were then attached to one ring proximally. Distally, an antero-posterior half pin was inserted medial to the tendon of the tibialis anterior, taking care to avoid the greater saphenous vein. A frontal plane Ilizarov wire was passed close to the ankle joint. These were then attached to the distal ring. To improve stability of the construct an empty ring was introduced in the center. A hybrid Ilizarov construct was used in all cases.

### Femoral Operative Procedure

**Figure 1:**
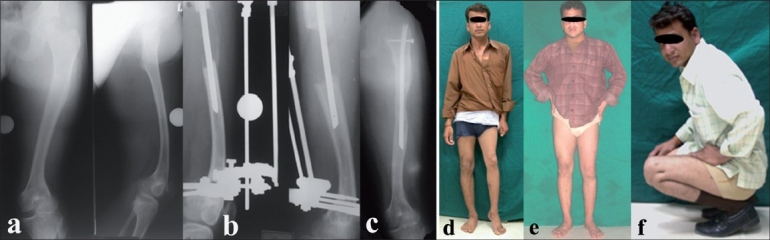
(a) Preoperative AP and lateral X-rays of left thigh including hip and knee. (b) Lengthening performed over a modified Humerus nail 7.5 cm length achieved. External fixator was removed in 13 weeks. (c) AP X-ray of left thigh showing bone consolidation with nail in situ. (d) 22 year old male with 8 cm shortening due to growth arrest following childhood septic arthritis of the lower femoral physis. (e,f) Final clinical result showing limb alignment and knee range of motion

In the femur all IM nails were introduced antegrade from the trochanter or piriformis fossa. The corticotomy site was pre-drilled; the femur was reamed 1.5 mm larger than the nail diameter. The risk of infection spreading from external fixation pins to the IM nail is greatest in the proximal femur. Here the pins are placed in the region of the lesser trochanter posterior to the nail with the cannulated drill technique.^1^ Distally, there is adequate room to pass the pins freehand to keep safe distance from the nail [Figure 1 a-f].

In a second surgery the IM nail was locked distally after completion of lengthening and the external fixation device was removed. All patients were given a plaster cast initially for a few days and then a customized molded removable brace to augment stability. This was done as an ample precaution, to compel the patient to be careful and to augment rotational stability.

Intravenous antibiotics were used for 48-96 h. Meticulous pin site care was maintained to ensure that no infection spreads to the marrow canal. Oral antibiotics were started at the first sign of pin track infection along with twice daily dressings. Local injection of antibiotics (125 mg of Injection Cefotaxim diluted in 1 ml saline, injected through the pin site and around it) had to be performed in two pins. None of these infections persisted or resulted in deep sepsis.

Fourteen associated procedures were performed in 11 patients. Five had a correction of equinus and four had a supracondylar osteotomy performed with an Ilizarov fixator. Four patients had a fixed flexion deformity of the knee joint ranging from 10 to 25° due to quadriceps paralysis following poliomyelitis which caused a hand to knee gait. This deformity was corrected with a supracondylar osteotomy performed percutaneously and fixed with an Ilizarov fixator.^4^ Two tibiae had correction of varus bowing. One had an ankle and subtalar fusion performed with the same modified device to achieve lengthening of 6 cm in the distal tibia. One patient with polio had a 6.8 cm femur lengthening with extension of the Ilizarov apparatus below the knee to correct a 40° fixed flexion deformity of the knee. One patient with hip dysplasia (case 27) had an Ilizarov hip reconstruction in the femur with a double level osteotomy and a tibial lengthening over a nail in which he achieved 6.4 cm of length.

## RESULTS

Goals of lengthening were achieved in all tibiae. In the femur, premature consolidation of the regenerate did not permit achievement of the full length in two cases. One premature consolidation was dealt with by a repeat corticotomy with the nail *in situ*.

External fixation period ranged from three weeks for a 1.5 cm lengthening to a maximum of 32 weeks for a 9.8 cm lengthening, with a mean of 12.9 weeks for a mean lengthening of 4.6 cm. Mean external fixation period was 17.8 days per cm.

There were no significant pin track infections. There were no deep intramedullary infections either early or late. Follow-up has ranged from three months to 12 years.

### Complications

Based on the system of Paley,^5^ there was one problem, three obstacles which required surgery and one minor and three major sequelae which persisted after treatment. The one problem was seen in one patient who achieved 5.5 cm of tibial length with correction of bowing. He developed an internal rotation deformity due to faulty fixation of the frame on the table. This was easily corrected with a gradual derotation construct in the Ilizarov fixator without the need for anesthesia or further surgery.

Of three obstacles which needed surgical correction, the first was a delayed union with poor regenerate formation in our first case which needed iliac crest bone grafting. The second was a persistent equinus contracture in a patient who achieved 9.8 cm of length, which needed a percutaneous tendoachilles (TA) Lengthening. The third was a premature consolidation of the femur. A repeat corticotomy was done with the nail *in situ*.

There was one minor and three major complications which persisted as sequelae. One adult patient developed a 10° procurvatum deformity in the proximal tibia. This was uncorrected, but was minor and had no effect on the function as there was good quadriceps strength. One major sequel was a significant varus angulation of 17° in a case of chondrometaphyseal dysplasia in which 8.5 cm of length was achieved. This was treated by removal of nail and complete correction with a Taylor's Spatial Frame fixator.^6^

**Figure 2:**
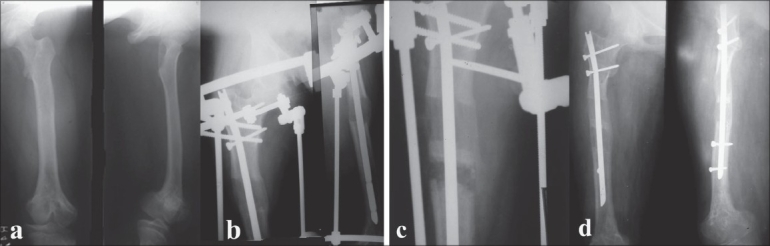
25 year old lady had 12 cm shortening of her femur due to growth arrest. (a) AP and lateral X-rays of thigh including hip and knee. (b) LON method with rapid bone formation lead to premature consolidation at 3 cm. (c,d) A repeat corticotomy was done with the nail in situ which allowed lengthening till 6 cm when a repeat premature consolidation did not allow equalization of limb lengths. Part of the problem was also with the low initial length of the nail which could not have permitted more length

The second was a precocious consolidation of the femur at 2.5 cm of lengthening. The patient had a residual shortening of 8 cm. The third was a repeat episode of premature consolidation in the patient who had had a successful repeat corticotomy [Figure 2]. She gained an additional length of 3 cm after the corticotomy and consolidated again despite increasing the rate of distraction. She has a persistent shortening of 6 cm.

There were no neurovascular complications. All femora (lengthening range 1.5 cm to 7.5 cm) retained full ROM in the knee. Time taken to regaining knee ROM ranged from four to 18 weeks. Regenerate formation was good in 26 cases and only in the first tibia lengthening over a nail performed by us an iliac crest bone graft was inserted in the regenerate site for improving the quality of the regenerate.

The Paley score^1^ of difficulties of lengthening was modified by us to take into account that most of our lengthenings were in the tibia which we found to range from a minimum of 4 to a maximum of 15 points with a mean of 7.6 points.

## DISCUSSION

The principles of limb lengthening elucidated by Ilizarov GA^7^,^8^ have made the Ilizarov technique scientific and reliable. The success rates of limb lengthening at the Ilizarov Institute in Kurgan have been partly replicated in many other centers including that of the author.^9^ However, the prolonged fixator duration tests the patience of the patient and when applied to the femur, this prolonged duration tends to increase knee stiffness.^1^

Moreover, eccentric location of bones in the limb creates a great tendency for axial deviation of the regenerate. This necessitates great care of the fixator during the lengthening phase as well as in the consolidation phase. Loosening of the fixation pins, instability of the fixator and tightness of muscles increase the tendency for axial deviation in the lengthening phase.

Many factors can delay the corticalization of the regenerate and prolong the duration of the external fixation. A traumatic corticotomy with displacement of bony ends, inadequate walking or instability of the frame, poor periosteal circulation as in polio, can all delay consolidation of the regenerate and removal of the fixator.

Combining IM fixation with the Ilizarov fixator firstly adds to the stability of the construct.^10^ This counteracts any adverse effect that reaming may have on regenerate formation.^11^ The next benefit of the addition of the IM nail is the prevention of axial deviation during the fixation period and also after removal of the fixator over the long term. This was proved in our series with very low rates of axial deviation. To achieve proper axial alignment of fragments in this method, care needs to be exercised in choosing the entry point of the IM nail. A lateral entry point in the upper tibia or upper femur will predispose to varus angulation and a medial entry point will predispose to valgus angulation. Deviation can also be caused by a narrow nail at the flared meta-diaphyseal junction, which is subjected to excessive muscular forces. This can be prevented by adding Poller screws.^12^ A posteriorly placed screw in the upper tibia can prevent procurvatum. This is crucial in polio as even a few degrees of procurvatum can mimic a fixed flexion deformity of the knee and lead to a hand-to-knee gait.^4^

This combined method is most beneficial for reducing duration of external fixation. Most patients are very happy with the early removal of the fixator and easily tolerate the discipline of crutches and partial weight-bearing and braces that is needed in the fixation period. We achieved a significant reduction in the external fixation duration in most of our patients to achieve mean external fixation duration of 17.8 days per cm. This too would seem fairly long as one may logically expect only 10 days per cm as the external fixation device should ideally be removed immediately after achievement of the length.

There are several reasons in our series for this extended period. Fourteen of the 27 segments had polio, a disease in which we frequently prefer to lengthen only at ½ mm per day instead of 1 mm per day. This is not only to accommodate for poorer regenerate formation but also to prevent contractures of the ankle with a higher rate of distraction.

The second reason is that one day per week no distraction is performed to allow dissipation of soft tissue tension. In a large lengthening like the 9.8 cm lengthening, there would be a rest day after every 6 mm (if done at 1 mm per day) or after every 3 mm (if done at ½ mm per day) as well as an initial latency of seven days. This would add at least 21 to 35 days more. In this patient we had used a modified Humerus nail (which originally had only one locking hole proximally and one distally) in which we failed to customize an additional distal locking hole. The shorter initial length of the tibia along with the significant lengthening meant that there was a short segment of the locked nail with only one screw below the regenerate. To prevent instability, we kept the external fixator on for much longer to allow some hardening of the regenerate.

A similar problem of customizing nails occurred in four other patients, where we chose to keep the fixator on for a longer duration. In many patients a lesser rate of lengthening ensured less pain as well as prevented and dissipated contractures of the hip and knee in femur lengthening, and knee and ankle in tibial lengthening. In the latter part of the series adequate customization of the nails with sufficient number of locking holes proximally and distally was done and we could remove the external devices sooner.

The greatest risk in this technique is IM infection due to close proximity of external pins and the nail. With great care in pin insertion and postoperative care we have not faced this problem in our series. However, the literature has mentioned this problem in the series of Simpson^11^ who had three deep infections in 20 LON surgeries. Kristiansen^13^ reported giving up on this method to revert back to the Ilizarov technique due to many complications.

The complication rate in our series compares very favorably with that reported by Kocaoglu.^14^ In their series reported in 2004, 18 complications occurred in 16 (38%) of the 42 segments for a rate of 0.43 complication per segment. There were two problems, 13 obstacles and three sequelae including one deep IM infection. In all they needed 16 surgeries to solve these 13 obstacles and three sequelae. These surgeries included three bone graftings, two tendon lengthenings, three repeat corticotomies, change of IM nail, change of fixator, surgical debridements etc.

In our series, we had 29.6% complications (eight in 27 lengthenings). There was one problem, three obstacles and three sequelae. We had to perform only four surgeries - two major (deformity correction with Taylor Spatial Frame (TSF)fixator, iliac crest bone grafting) and two minor (percutaneous TA Lengthening, repeat corticotomy). One of our sequelae was minor - a deformity of 10° tibial procurvatum, which did not interfere with function. One patient developed a premature consolidation of the femur, which left him 8 cm short. Most important, we did not have any deep medullary infection as compared to one out of 42 in Kocaoglu's series and three out of 20 in the series of Simpson (15%). However, two of the three infections in Simpson's series had shortening due to compound fractures and previous infection.

Sixteen cases in our series had a Paley difficulty score of more than 6.5; 11 segments were lengthened to 6 cm or more, and percentage lengthening of 21.5% or more was performed in seven of our cases-these being the criteria as suggested by Kocaoglu to indicate a higher incidence of difficulties and propensity for complications. Hence our rate of complications is comparatively lesser as compared to the level of difficulties.

Most certainly the future will see many lengthening surgeries being performed only with the help of fully implantable IM devices without the need for external fixation. This may be possible in cases without severe deformities or contractures. Such devices are available and have a mechanical control as used by Guichet^15^ in the Albizzia nail or as used by Baumgart^16^ in the electronically controlled FITBONE device. These are, however, not yet easily available or affordable.

At present, a combination of internal and external fixation technique does have a role in safely reducing axial deviation as well as duration of fixation.

## SUMMARY

Lengthening over an IM nail is a useful procedure in reducing the duration of external fixation as well as reducing chances of axial deviation in limb lengthening. Risks of IM infection are real and should be prevented by meticulous technique.
